# Effects of Polyhalite Fertilization on Skin Quality of Potato Tuber

**DOI:** 10.3389/fpls.2019.01379

**Published:** 2019-10-30

**Authors:** Alexandra Keren-Keiserman, Ravi Singh Baghel, Edna Fogelman, Inna Faingold, Uri Zig, Uri Yermiyahu, Idit Ginzberg

**Affiliations:** ^1^Institute of Plant Sciences, Agricultural Research Organization, Volcani Center, Rishon LeZion, Israel; ^2^Institute of Soil, Water and Environmental Sciences, Agricultural Research Organization, Gilat Research Center, Negev, Israel; ^3^Hevel Maon Enterprises, Negev, Israel

**Keywords:** periderm, polyhalite, potato skin, russeting, skin blemish, *Solanum tuberosum*

## Abstract

The protective peel of potato tuber consists of periderm tissue, the outmost cell layers of which contain corky cell walls and are termed “skin”. The skin protects the tuber from water loss and pathogen invasion, and its visual appearance is a highly important marketing factor. Physiological skin blemishes are of great concern, mainly russeting disorder and skinning injuries. We previously showed that application of calcium (Ca) reduces the rate and severity of skin russeting. Here, polyhalite fertilization was tested as an alternative source of Ca. The polyhalite mineral is a hydrated sulfate of potassium (K), Ca, and magnesium (Mg), and thus contains additional important nutrients that may contribute to skin quality. Furthermore, in view of the direct interaction of soil mineral elements with the tuber skin, we tested application of polyhalite at the end of the growth period, assuming that providing the mineral at the last stages of skin development may enhance its quality. Accordingly, polyhalite was applied at three time points: preplanting, in-season at around 3–4 weeks prior to haulm desiccation, and 2 days post-haulm desiccation. The experiments included several cultivars and locations. Data indicated that late application of polyhalite, after haulm desiccation, results in reduced concentrations of Ca and Mg and increased concentration of K in the tuber peel of fertilized plants compared to controls. Tuber appearance was improved, and the expression of *FHT* and *CYP86A33*, indicator genes for skin suberization, was significantly upregulated. Earlier applications of the polyhalite mineral did not alter mineral elements concentrations in the tuber peel compared to control plants. Overall, polyhalite fertilization positively affected tuber skin appearance and skin-related gene expression. However, the effect was moderate, and the mineral did not fully mitigate skin imperfections. The effect of polyhalite may be dependent on local conditions and cultivar type.

## Introduction

The marketability and price of fresh market potatoes are determined by the tuber’s visual appearance, which is largely a function of tuber shape, color, and skin. Smooth-skinned varieties are characterized by a clean, shiny appearance compared to the earthy look of russeted potatoes, where soil often adheres to the rough skin surface. The rough skin of russeted varieties (e.g., the well-known US variety Russet Burbank) is a desired, genetically inherited character (de Jong 1981). However, russeting of smooth-skinned varieties often occurs under suboptimal growth conditions (Ginzberg et al., 2009), and these potatoes are rejected by the consumer.

Physiological skin blemishes that are not caused by pathogens, mainly the russeting phenomenon and skinning injuries, are therefore of great concern (Ginzberg et al., 2005; Ginzberg et al., 2009; Ginzberg et al., 2012; Fogelman et al., 2015; Vulavala et al., 2016). In potato, russeting refers to dead skin cells that remain adhered to the newly formed skin layers below them (Ginzberg et al., 2012); during normal skin development, these cells are sloughed off, rendering the skin smooth and shiny. Skinning injuries refer to mechanical wounding of the skin during harvest that detaches the skin from the tuber flesh due to problems in skin maturation (skin-set) (Lulai 2002). In addition to the marketing value of tuber appearance, skin quality is an important factor in preventing tuber water loss and pathogen invasion, and consequently, stored tuber quality. We previously suggested that although potato tubers are considered to be low-transpiring organs as they are surrounded by moist soil, russeted skin can enhance tuber water loss (Ginzberg et al., 2012).

The potato skin is the outermost layer of the tuber periderm, a protective tissue of secondary origin that replaces the epidermis when the latter is damaged. The periderm is made up of three cell types: phellem, phellogen, and phelloderm (Reeve et al., 1969). The phellem (or cork) forms a series of layers at the outermost level of the periderm, and is derived from the meristematic phellogen layer (or cork cambium) below it. As phellem cells develop, they become suberized and then die, forming a protective layer termed “skin”. The phelloderm cells form the innermost layers of the periderm, and are similarly derived from the phellogen layer, which is located directly above them. New skin layers are continuously added by cell division during tuber expansion, whereas superficial cork cells are sloughed off, rendering the skin smooth and shiny.

Suberin, which accumulates in the phellem cell walls, is a macromolecule containing both polyaromatic and polyaliphatic domains that is found between the primary cell wall and the plasma membrane. The aromatic domain is composed of monolignols and hydroxycinnamic (ferulic) acids and is covalently bound to the primary cell wall (Bernards and Lewis 1998; Yan and Stark 2000). The aliphatic domain is a polyester that, upon transesterification, releases mainly C16–C28 α,ω-diacids and ω-hydroxyacids, with minor amounts of alkan-1-ols, alkanoic acids, and glycerol. The latter may be involved in cross-linking between the aromatic and aliphatic domains (Graça and Pereira 2000; Graça 2015).

The suberin-biosynthesis pathway has not been completely resolved; it includes β-ketoacyl-coenzyme A (CoA) synthases (KCS), fatty acyl reductases, long-chain acyl-CoA synthetases, cytochrome P450 monooxygenases, glycerol 3-phosphate acyltransferases, and phenolic acyltransferases (reviewed in Vishwanath et al., 2015). Most studied in potato are: StKCS6 which catalyzes suberin and wax compounds with chain lengths of C28 and longer (Serra et al., 2009a), the cytochrome CYP86A33 which has been shown to promote the ω-hydroxylation step and its silencing, leading to a reduction in aliphatic suberin load and increased permeability of the periderm (Serra et al., 2009b), and feruloyl transferase (FHT), suggested to ester-link ferulic acid to ω-hydroxyacids and fatty alcohols into potato suberin (Serra et al., 2010).

We previously showed that application of calcium chloride reduces the rate and severity of skin russeting (Ginzberg et al., 2012). The effect of calcium (Ca) on the skin could originate from transport of Ca with the transpiration stream *via* the xylem into the tuber, or from direct interaction of the skin with the soil solution, which surrounds the tuber. Being surrounded by moist soil, potato tubers are considered low-transpiring organs, and therefore prone to Ca deficiency (Palta 2010). Thus, direct interaction of the tuber skin with Ca in the soil solution may be the main reason for the beneficial effect of Ca on the skin. Accordingly, the formula used to apply Ca to the soil may affect its interaction with the skin at the surface of the tuber and its availability to the plant. The exact effect of Ca on potato skin is not clear, although it has been implicated in maintaining the structural integrity of cell walls and intracellular adhesion (Vincken et al., 2000; Jarvis et al., 2003). Note that other divalent ions in the soil extract, such as silica, may also interact directly with the skin (Vulavala et al., 2016).

There are various formulas and modes of application for Ca fertilization. Here, we made use of a fertilizer produced from the mineral polyhalite. Polyhalite [K_2_Ca_2_Mg(SO_4_)4·2H_2_O] is a hydrated sulfate of potassium (K), Ca, and magnesium (Mg) in the relative proportions: 48% sulfur trioxide (SO_3_), 17% calcium oxide (CaO), 14% potassium oxide (K_2_O), and 6% magnesium oxide (MgO), and as such contains additional important nutrients that may contribute to skin quality. Furthermore, in view of the direct interaction between soil minerals and the tuber skin, we tested polyhalite application at the end of the growth period, assuming that providing the mineral at the last stages of skin development might enhance the tuber’s market value.

## Materials and Methods

### Soil, Climate, and General Growth Management

The experiments were carried out in the Western Negev region of Israel which is characterized by a semiarid Mediterranean climate, with average annual rainfall of 200–300 mm during the winter (November–March), and dry summers.

Experiments were conducted in two soil types: sandy clay loam (clay 20%, silt 7.5%, and sand 72.5%, w/w), and fine sand (clay 8.5%, silt 3.5%, and sand 88%, w/w). Essential elements composition of water extract of the soils (1:1 w/w): Ca, 0.7–1 mM; Mg, 0.1–0.3 mM; and K, 1.5–2.0 mM.

Irrigation (sprinklers) was with reclaimed water, with the following major components (mg/L): chloride, 182; sodium, 133.5; Ca, 83.6; sulfur, 23.88; Mg, 18.62; K, 11.1; phosphorus, 0.155; and pH 7.69. Irrigation was applied every 3–4 days, 200 m^3^/ha. Total amount of water was around 4,000–4,800 m^3^/ha, depending on growth period and occasional rain. Following haulm desiccation, irrigation was applied at 70 m^3^/ha every 5–7 days – that is to keep the soil moist for prevention of surface cracking and penetration of pests into tuber region, and to allow optimal skin-set. When polyhalite was applied after haulm desiccation, it was followed by water irrigation of 250 m^3^/ha.

Beds were of two ridges with total width of 1.93 m. Each experiment described below was done in 5–6 replicates, 20 m bed length for each replicate. Replicate blocks were organized in random. Growth practices included preplanting application of 20 m^3^/ha compost, followed by sprinkler irrigation of 250 m^3^/ha. From 40 to 80 days after seed-tuber planting (DATP), nitrogen (N) was applied at 320 kg/ha as ammonium nitrate by fertigation; 20–30 units of N every irrigation. N concentration in the plant was monitored by petiole test. Only for fine sand soil, phosphate was applied preplanting together with the compost, at 200 units/ha. Haulms were desiccated, as specified in each experiment below, with Reglone^®^ (Syngenta, Basel, Switzerland).

### Experimental Design and Polyhalite Application

*Experiment 1*: The experiment was performed with the red-skinned potato cv. Rosanna which is prone to severe russeting and skinning injuries. Tubers were planted in January 2016 in a commercial field with sandy clay loam soil. Polyhalite in the form of Polysulphate^TM^ product (http://www.polysulphate.com/fertilizing-potatoes-with-polysulphate/) (2–4 mm granular product, produced by ICL at Boulby Mine in the UK) was applied preplanting at 1,500 kg/ha, followed by sprinkler irrigation (PolyH plants). Control plants were not fertilized with the polyhalite (C plants). Late application of Polysulphate (1,500 kg/ha in powder form) was performed manually on bed tops at the end of growth (120 DATP; 2 days after haulm desiccation), when the foliage was partially wilted, followed by sprinkler irrigation. This late polyhalite treatment (PolyH” plants) was applied to half of the beds of PolyH plants, and to half of the beds of their controls. Accordingly, at harvest, sampling included tubers from four types of plants: the C plants that were not fertilized with polyhalite at all, C+PolyH” plants that were fertilized only at the late application, PolyH plants that were fertilized only at the beginning of growth, and PolyH+PolyH” plants that received polyhalite at the beginning and end of the growth period. Experimental design included six replicates arranged in randomized blocks for each polyhalite treatment. Tubers were collected at the late tuber-bulking stage (100 DATP), at tuber maturation, when haulm desiccation was applied (118 DATP), and at harvest (135 DATP). At each time point, treatment, and replicate, tubers were collected from 1–1.5 m of bed (8–10 plants, 70–100 tubers), and the 10 biggest tubers were selected for further analyses. Tuber skin was sampled from all 10 tubers, hence each sample (for time point, treatment and replicate) represented several plants. Skin samples were used for mineral elements analysis, and extraction of RNA for molecular analysis of skin reporter genes. Visual evaluation of tuber appearance was performed at harvest and after 1 month of storage.

*Experiment 2*: This experiment tested the effect of polyhalite application only at the end of growth on tuber visual appearance. It was performed in different plots, in sandy clay loam soil. Polysulphate powder was applied at 1,500 kg/ha 2 days after haulm desiccation, followed by sprinkler irrigation, as in Experiment 1 for the PolyH” plants. The experiment was performed with the early (winter growing) white-skinned potato cv. Arizona that is prone to russeting and skinning injuries, and three late potato cultivars (spring growing)—two white-skinned cultivars, Sifra and Panamera, and red-skinned cv. Mozart. Experimental design included five replicates arranged in randomized blocks for each cultivar. Visual evaluation of tuber appearance was performed after 1 month of storage.

*Experiment 3*: The experiment was performed with two potato cultivars at two locations. The red-skinned cv. Rosanna in a sandy clay loam soil, and the white-skinned cv. Vivaldi in a fine sand soil. The latter also exhibits skin russeting, albeit to a lesser extent than Rosanna. Polysulphate (1,500 kg/ha, granular) was applied preplanting of seed tubers at the end of October 2016. In-season application of Polysulphate powder (1,500 kg/ha) was performed manually on bed tops at 85 DATP for Rosanna and 95 DATP for Vivaldi, followed by sprinkler irrigation. Haulm desiccation was applied around 3–4 weeks later. Tubers were harvested in mid-March 2017, 140 DATP. Experimental design included five replicates arranged in randomized blocks. Potatoes were collected at early tuber-bulking stages, 60 and 75 DATP, and at the late tuber-bulking stage, 95 DATP for Rosanna, and 105 DATP for Vivaldi. Tubers were also collected at harvest (140 DATP) for visual evaluation. For each cultivar, at each time point, treatment and replicate, tubers were collected from 1–1.5 m of bed (8–10 plants, 70–100 tubers), and the 10 biggest tubers were selected for further analyses. Tuber skin was sampled from all 10 tubers, hence each sample (for cultivar, time point, treatment and replicate) represented several plants. Skin samples were used for mineral elements analysis, anatomical study, and the extraction of RNA for molecular analysis of skin reporter genes. Visual evaluation of tuber appearance was performed after 2 months of storage.

### Tuber Storage Conditions and Assessment of Physiological Skin Blemishes

At the end of the growth, after harvest, tubers were stored at 14°C for 2 weeks (curing), followed by a gradual decline in temperature to 2°C, and were stored at 2°C for 1–2 months, as practiced in the potato industry. After storage, tubers were acclimated to 6–8°C for a week, then washed, allowed to dry for 24 h at room temperature, and evaluated for skin appearance. Twelve tubers for each treatment and biological replicate were compared visually to a reference cultivar, using a visual index of 1–5, with 5 indicating high skin quality and 1, poor skin quality. The evaluation panel included four people who rate commercial products routinely.

### Determination of Mineral Elements in the Tuber Periderm

Samples of tuber skin were dried in an oven at 60°C and pulverized. Total concentrations of K, Ca, and Mg were determined by atomic absorption spectrophotometry (Perkin-Elmer Model 460) after digestion with nitric acid and perchlorate for Ca and Mg, and with sulfuric acid and peroxide for K (Snell and Snell 1949). Mineral elements concentration in the plant tissues was calculated as percentage of dry matter.

### Skin Anatomical Study

Samples of the tuber surface (blocks of 4 x 3 x 3 mm) were fixed in FAA (50% ethanol, 5% acetic acid, and 3.7% formaldehyde, v/v), dehydrated in an ethanol/Histoclear (Finkelman Chemicals, Petach-Tikva, Israel) series and embedded in paraplast (Paraplast Plus, McCormick Scientific, St. Louis, MO) according to standard methods (Ruzin 1999). Tissue sections (15–20 µm) were stained with Safranin-O/Fast green (Sigma Chemicals, Rehovot, Israel) for examination of tissue morphology (Johansen 1940). Sections were observed under a light microscope (Leica DMLB, Wetzlar, Germany) and images were displayed on a monitor through a CCD camera (Leica DC2000) using the Leica IM1000 program. The same samples were viewed under UV light to detect autofluorescence of suberized cell walls in the skin: the Leica DMLB microscope was configured for epifluorescent illumination using an HBO103W/2 mercury lamp, excitation filter BP 340–380, chromatic beam-splitter FT 400 and barrier filter LP 425.

### RNA Extraction and Quantitative Real-Time PCR (qPCR)

Total RNA was extracted according to Ginzberg et al. (2009). cDNA was synthesized from total RNA using EZ-First strand cDNA Synthesis kit for qRT-PCR (Biological Industries, Beit Haemek, Israel), and ABsolute^TM^ Blue QPCR SYBR^®^ Green ROX Mix (Thermo Scientific, Waltham, MA) was used for qPCR according to the manufacturer’s protocol with specific primers (**Table 1**). Each qPCR was performed with three biological replicates (out of five or six biological replicates that were sampled for each cultivar, time, and treatment), each with three technical replicates. Values in each sample were normalized to the expression levels of the α-chain of the nascent polypeptide-associated complex (*α-NAC*, Sotub10g027110) as the reference gene (Ginzberg et al., 2009).

**Table 1:** Primers used in the study.

Gene	Sotub ID	Forward primer (5'→3')	Reverse primer (5'→3')	Amplicon size (bp)
*CYP86A33*	Sotub06g032570	TGGAGTTTAAACCGGAGAGA	TCAACTTTATGACCGGGAAC	199
*FHT*	Sotub03g018220	CTACTTGGTCTAGGCTTTC	CTGTTTAGATCTCCATAAGTTC	198
*KCS6*	Sotub02g029020	TACATCGAGGCCAAAGGA	ATCTCTGGGATGAACACTGG	173
*NAC*	Sotub10g027110	ATATAGAGCTGGTGATGACT	TCCATGATAGCAGAGACTA	97

### Data Analysis

Data were analyzed for statistical significance by two-factorial analysis of variance, or by Student’s *t*-test for one-factor analysis. Both analyses used JMP software (http://www.jmp.com). Significant difference was determined at *P* < 0.05.

## Results

### Polyhalite Fertilization and Mineral Elements Concentration in Tuber Peel – Experiment 1

**Figure 1:**
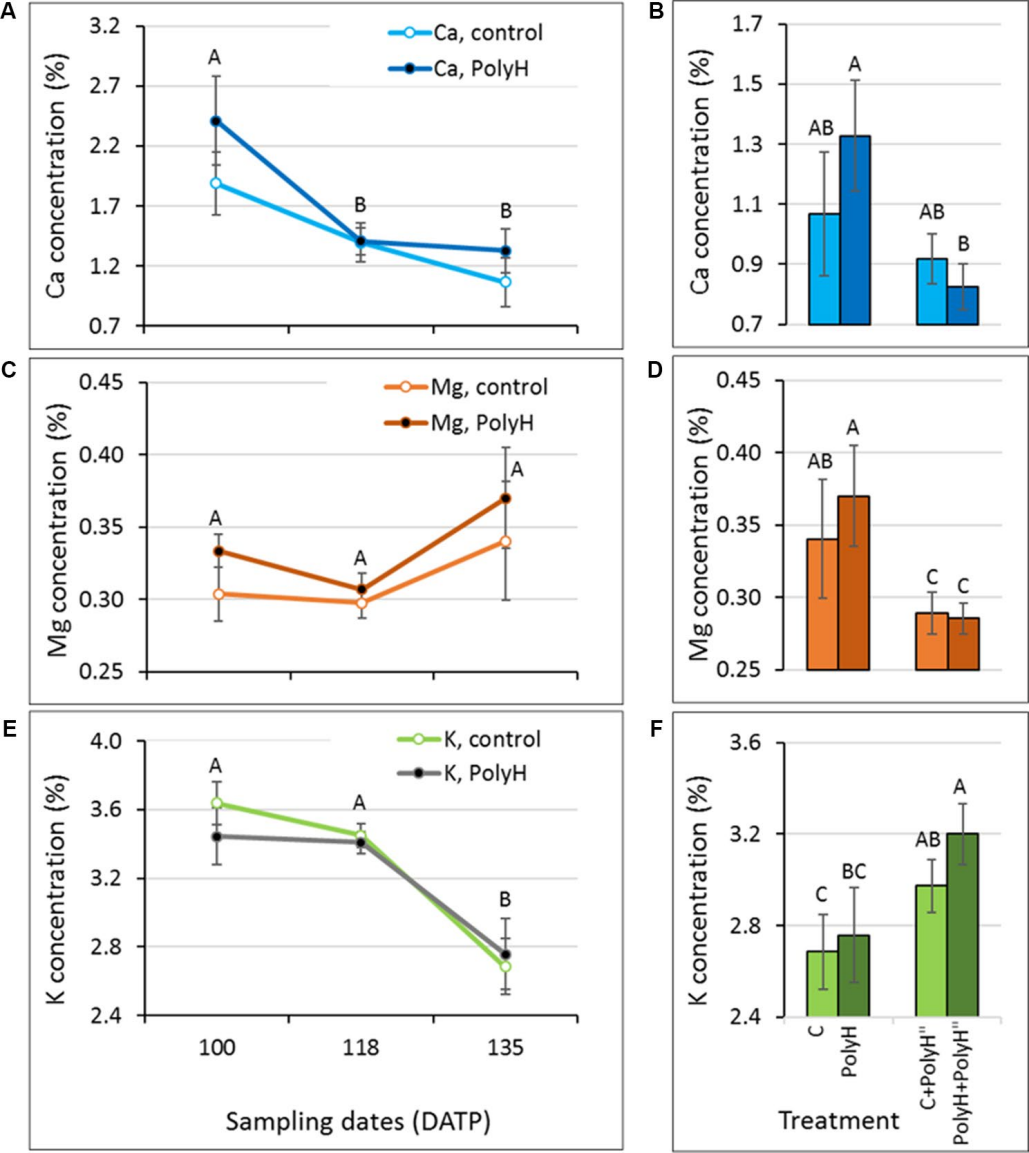
Ca, Mg, and K concentrations in the peel of maturing Rosanna tubers following polyhalite fertilization (experiment 1). **(A**, **C**, **E)** Potato plants were fertilized with polyhalite preplanting (PolyH); control plants (C) were not fertilized. Tuber peel was collected at 100, 118, and at harvest, 135 DATP. Haulm desiccation was applied at 120 DATP; hence, the skin-set process occurred between that time and 135 DATP. **(B**, **D**, **F)** Late application of polyhalite, 2 days after haulm desiccation, to both PolyH plants (PolyH+PolyH") and their controls (C+PolyH"). Peel samples were collected at 135 DATP together with the PolyH and C samples. Bar charts compare mineral elements concentrations in mature peel at 135 DATP following polyhalite application at time of planting and polyhalite application following haulm desiccation. Mineral elements concentrations are given as percentage of peel dry weight. **(A**, **B)** refer to Ca, **(C**, **D)** to Mg, and **(E**, **F)** to K concentration. Values are averages of six replicates with SE. Two-factor analysis—time and treatment—was performed with data of charts **(A**, **C)**, and **(E)**. No interaction was found. Different letters indicate significant difference by Student’s *t*-test (*P* < 0.05) between time points, with no difference between treatments. Data of charts **(B**, **D)**, and **(F)** were analyzed for statistical significance as one variable (treatment) by Student’s *t*-test; different letters indicate significantly different values (*P* < 0.05).

The level of mineral elements making up the polyhalite—Mg, Ca, and K—was monitored in tuber peel samples collected from Rosanna plants that were fertilized preplanting or following haulm desiccation, and their controls. The objective was to test whether polyhalite fertilization altered skin Mg, Ca, and K concentrations (**Figure 1**). The concentration of Ca and K decreased significantly from 100 DATP to harvest (135 DATP) in peels of both PolyH and C plants, and no significant difference was found between them at any time point (**Figures 1A, E**). An increase in Mg concentration during the skin-set process (between haulm desiccation at 118 DATP and harvest at 135 DATP) was noted, but it was not significant (**Figure 1C**). In general, at harvest (135 DATP), tuber peels from PolyH plants had slightly increased (but not significantly so) mineral elements concentrations (**Figures 1A, C, E**, point 135 DATP). When polyhalite was applied following haulm desiccation, K concentrations increased significantly in peels of both C+PolyH” and PolyH+PolyH” plants, compared to C and PolyH plants, respectively (**Figure 1F**). In contrast, concentrations of Ca and Mg decreased (**Figures 1B, D**). It could be concluded that polyhalite treatment following haulm desiccation altered the concentrations of the tested mineral elements in the tuber peel.

### Visual Evaluation of Tuber Skin Appearance – Experiment 1

**Figure 2:**
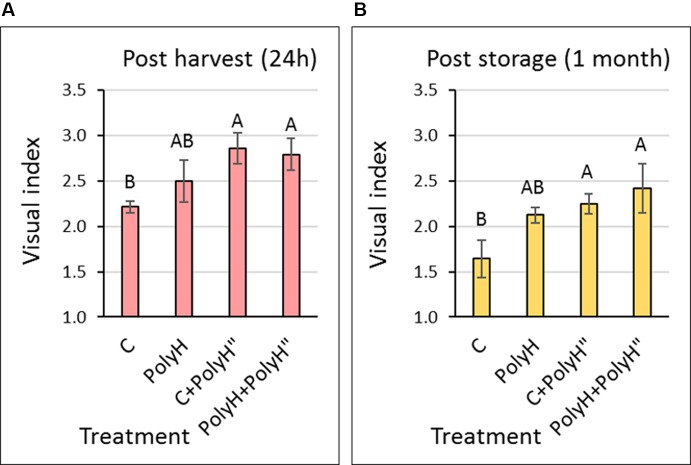
Visual evaluation of Rosanna tubers from experiment 1. Samples labeling is as described in legend of **Figure 1**. Visual evaluation of tuber appearance was performed 24 h after harvest **(A)** and after 1 month of storage **(B)**. Values are averages of six replicates with SE. Each replicate consisted of 12 tubers. Visual index was determined on a scale of 1 to 5: 5 = good skin quality, 1 = low skin quality, by a panel of four individuals who rate commercial products routinely. Data were analyzed for statistical significance by Student’s *t*-test; different letters indicate significantly different values (*P* < 0.05).

Tuber appearance was examined at two time points: 1 day after harvest (**Figure 2A**) and after 1 month in storage (**Figure 2B**). The late application of polyhalite following haulm desiccation (PolyH” treatment) significantly improved tuber appearance when compared to the nontreated control (C). This was true for tubers of C+PolyH” plants that were treated only at the late application, and for tubers from PolyH+PolyH” plants that received the polyhalite twice. This trend was the same whether tubers were evaluated immediately after harvest (**Figure 2A**) or following 1 month of storage (**Figure 2B**). However, overall quality of the skin was reduced during storage; this can be clearly seen for tubers from C plants whose visual index was reduced significantly in storage by 27% (sample C in **Figure 2A** compared to sample C in **Figure 2B**). Although late polyhalite treatment (PolyH”) improved skin appearance, the visual index values were lower (around 3) than the standard required for marketing (index value of 4–5).

### Skin Marker Gene Expression – Experiment 1

**Figure 3:**
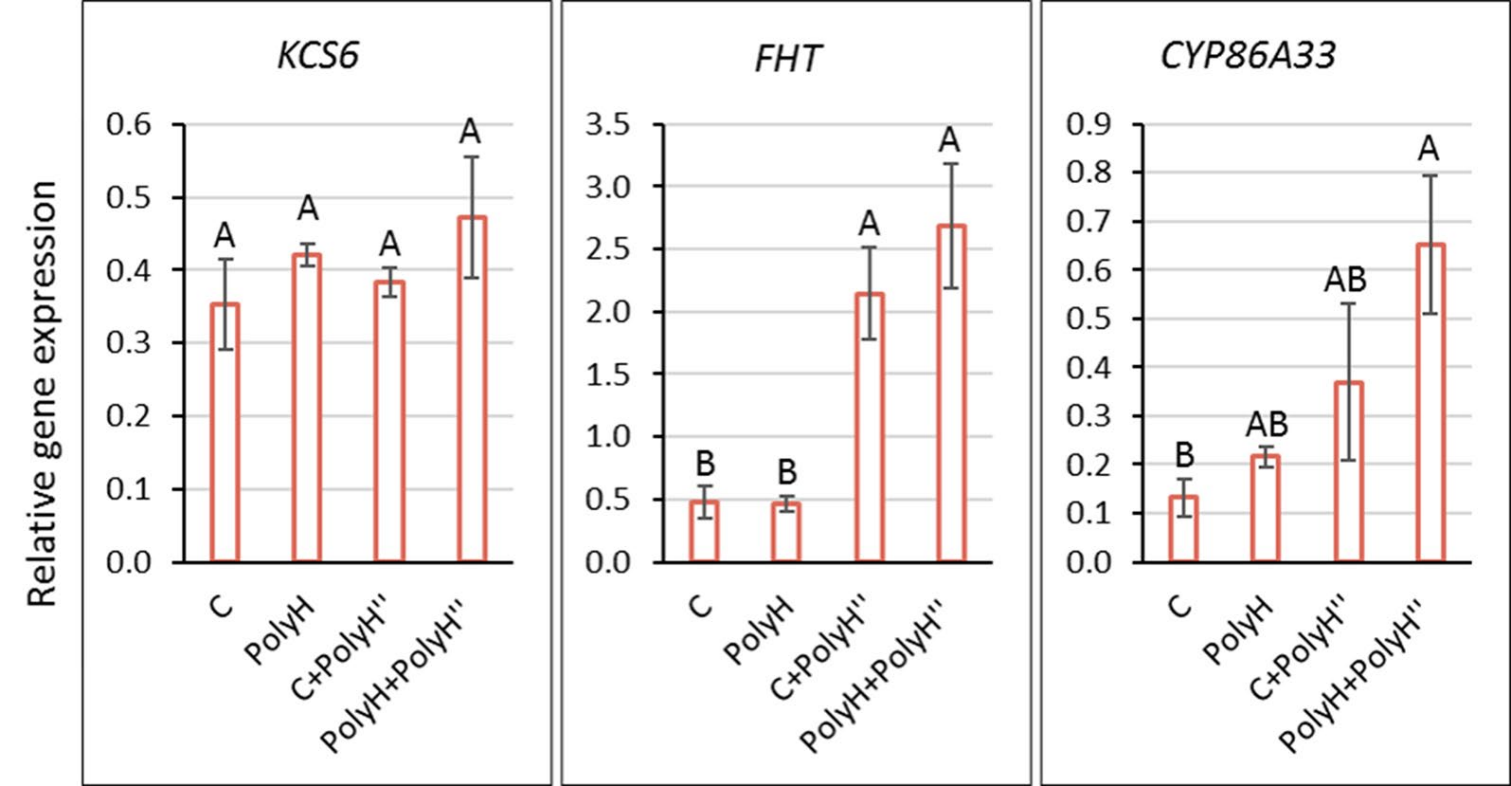
Expression of *KCS6*, *FHT*, and *CYP86A33* indicator genes for skin suberization in the peel of Rosanna tubers from experiment 1. Samples labeling is as described in legend of **Figure 1**. Peel samples were collected at harvest (135 DATP). Transcript levels were monitored by qPCR and expression levels were normalized relative to that of the reference gene α-*NAC*. Values are averages of three replicates with SE. Data were analyzed for statistical significance by Student’s *t*-test; different letters indicate significantly different values (*P* < 0.05).

To understand the observed positive effect on skin appearance, the expression of indicator genes for skin suberization was determined in tuber peel following the polyhalite treatments (**Figure 3**). *KCS6* did not exhibit differential expression between treatments. The expression of *FHT* and *CYP86A33* was higher in tuber peel following the late application of the polyhalite, i.e., C+PolyH” and PolyH+PolyH” plants compared to controls. Overall, late application of polyhalite following haulm desiccation affected two of the tested skin-related genes.

### Application of Polyhalite Only at the End of Growth – Experiment 2

**Figure 4:**
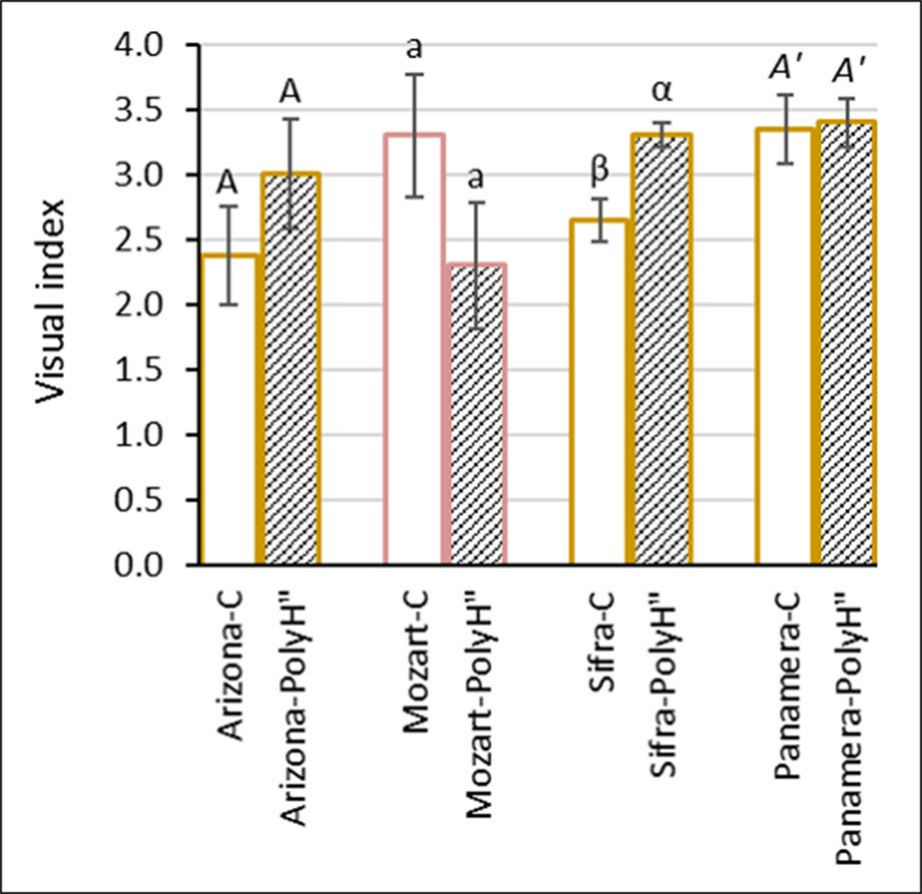
Evaluation of skin appearance of the early variety Arizona (white skin), and the late varieties Mozart (red skin), Sifra, and Panamera (both white skin) (experiment 2). Potato plants were fertilized with polyhalite 2 days after haulm desiccation (PolyH"); control plants (C) were not fertilized. Tubers were examined after 1 month in storage. Visual index and assessment were as described in **Figure 2**. Values are averages of five replicates. Data were analyzed for statistical significance by Student’s *t*-test for each cultivar separately. Different letters between the cultivar’s C and PolyH" samples indicate significantly different values (*P* < 0.05).

Overall data indicated a positive effect of polyhalite on tuber skin of Rosanna when applied at the end of growth following haulm desiccation (experiment 1); therefore, an additional experiment was conducted to test this approach with other potato cultivars. Experiment 2 was performed with the early (winter growing) white-skinned cv. Arizona which is prone to russeting and skinning injuries, and three late (spring growing) cultivars: white-skinned cvs. Sifra and Panamera, and red-skinned cv. Mozart (**Figure 4**). As in experiment 1, polyhalite was applied 2 days after haulm desiccation, and tuber appearance was evaluated after 1 month in storage. Results differed between the cultivars: late application of polyhalite significantly improved the skin appearance of cv. Sifra. However, no significant effect was observed for the other cultivars.

### Application of Polyhalite Before Haulm Desiccation, and Mineral Elements and Visual Analyses of Tuber Peel – Experiment 3

To maximize the positive effect of polyhalite on skin appearance, we hypothesized that its application a few weeks before haulm desiccation, while skin development is highly active, would enhance its effect. The experiment was performed with the red-skinned cv. Rosanna and the white-skinned cv. Vivaldi. The experimental design was similar to that in experiment 1, except that the second application of polyhalite was done in-season, 3–4 weeks before haulm desiccation. Accordingly, tuber peel was sampled at early time points in tuber development: during early tuber bulking, 60 and 75 DATP, as well as 10 days after the second polyhalite application, 95 DATP for Rosanna, and 105 DATP for Vivaldi.

**Figure 5:**
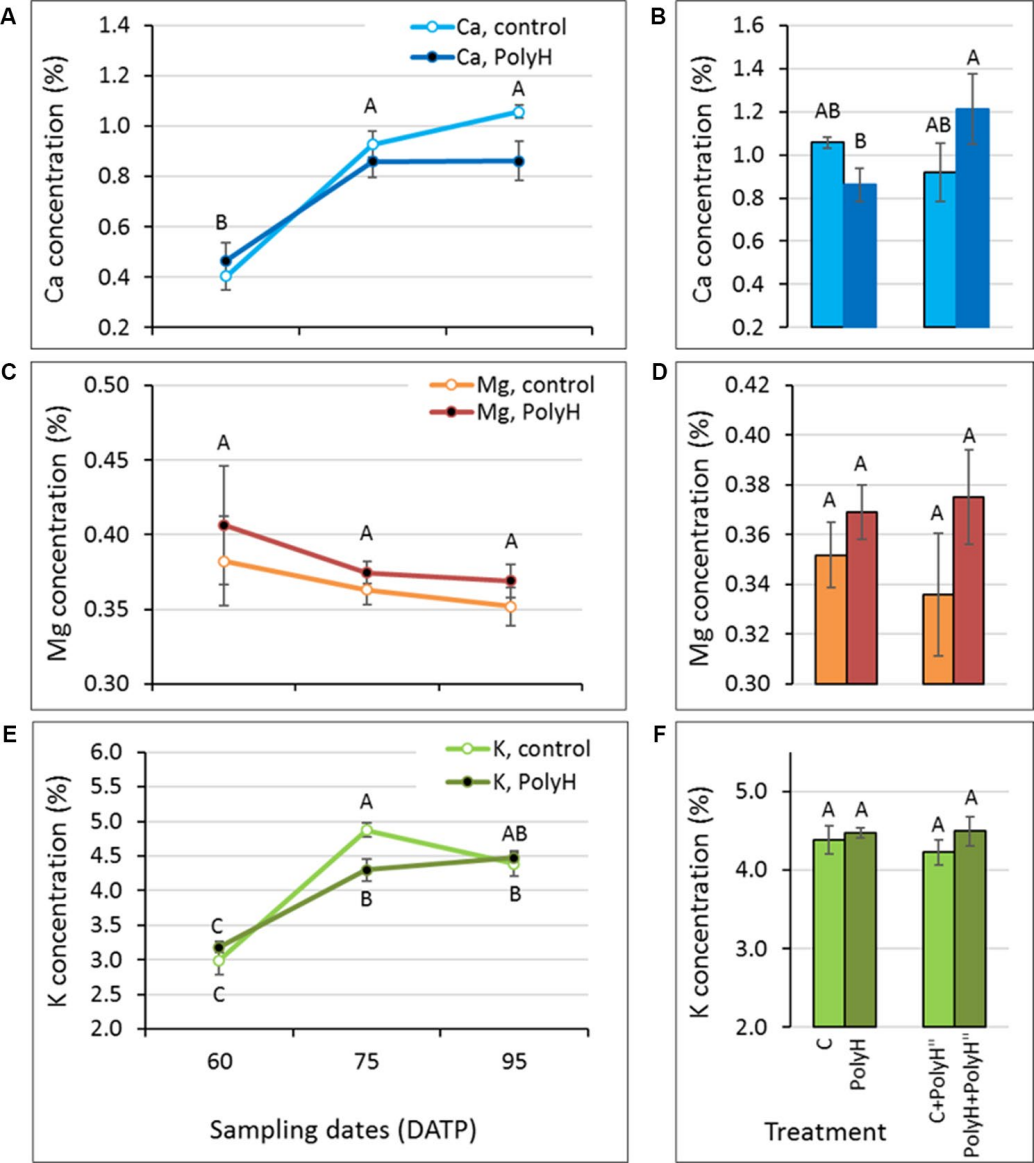
Ca, Mg, and K concentrations in the peels of early- and late-bulking Rosanna tubers following polyhalite fertilization (experiment 3). **(A**, **C**, **E)** Potato plants were fertilized with polyhalite preplanting (PolyH); control plants (C) were not fertilized. Tuber peel was collected at 60, 75, and 95 DATP. **(B**, **D**, **F)** Late in-season application of polyhalite (PolyH") was performed at 85 DATP for both PolyH plants (PolyH+PolyH") and their controls (C+PolyH"). Peel samples were collected at 95 DATP together with the PolyH and C samples. Bar charts compare mineral elements concentrations in tuber peel collected at 95 DATP following polyhalite application at preplanting and polyhalite application in-season. Mineral elements concentrations are given as percentage of peel dry weight. **(A**, **B)** refer to Ca, **(C**, **D)** to Mg, and **(E**, **F)** to K concentration. Values are averages of five replicates with SE. Two-factor analysis—time and treatment—was performed with data of charts **(A**, **C)**, and **(E)**. Interaction was found only for K concentrations. For Ca and Mg, different letters indicate significant difference by Student’s *t*-test (*P* < 0.05) between time points, with no difference between treatments. Data of charts **(B**, **D)**, and **(F)** were analyzed for statistical significance as one variable (treatment) by Student’s *t*-test; different letters indicate significantly different values (*P* < 0.05).

Following preplanting application of polyhalite, mineral elements analysis of tuber peel from Rosanna indicated significant increases in the concentrations of Ca and K between 60 and 75 DATP, with no significant change afterwards (**Figures 5A, E**). This pattern was evident for both C and PolyH plants. A slight but nonsignificant decrease was obtained for Mg concentration during skin development and following treatment (**Figure 5C**).

After the in-season application of the polyhalite, no significant differences were obtained for Ca, Mg or K concentrations when peels from C+PolyH” and PolyH+PolyH” plants were compared to their controls (**Figures 5B, D, F**).

**Figure 6:**
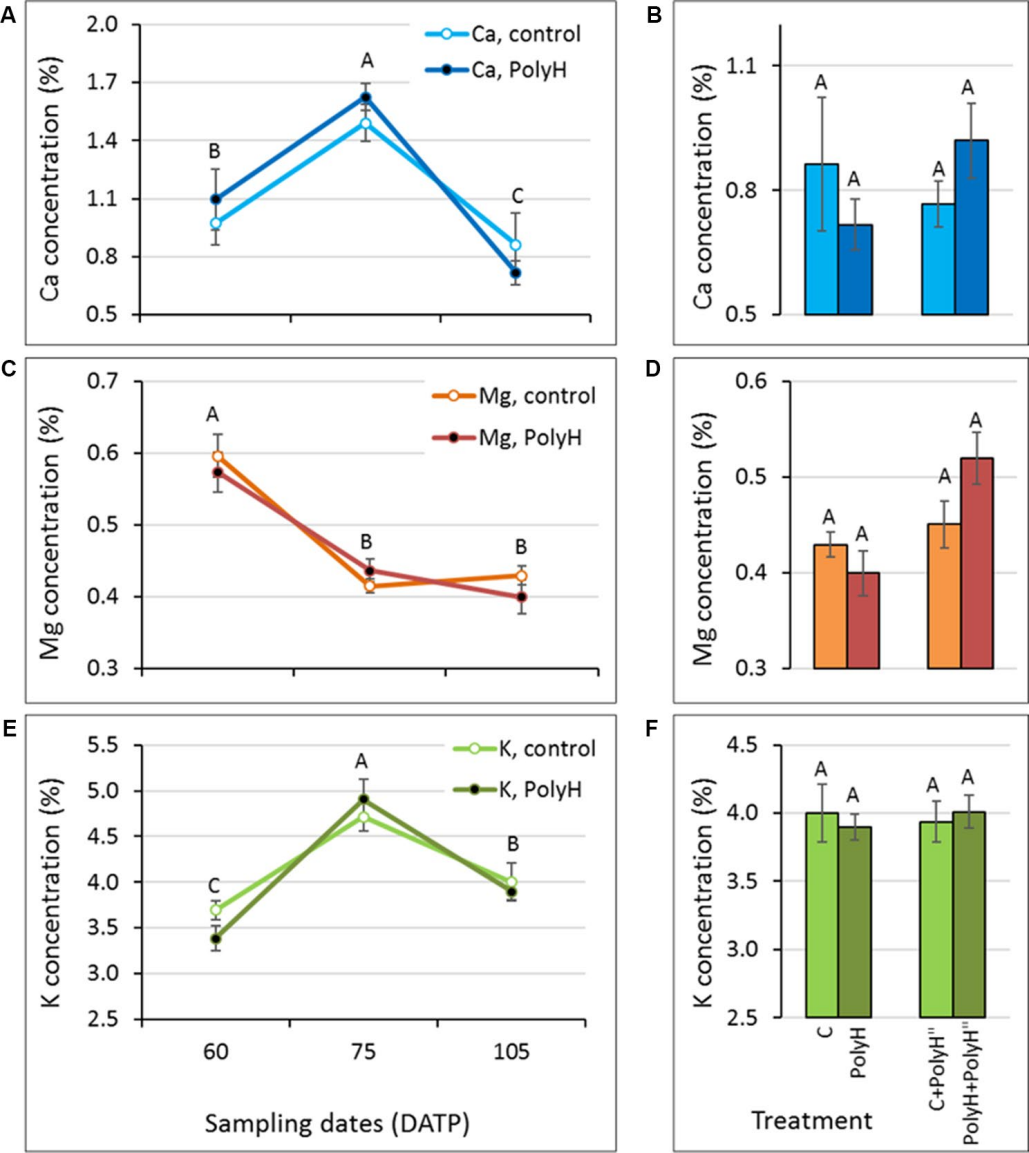
Mineral elements concentration in peels of early- to late-bulking Vivaldi tubers following polyhalite fertilization (experiment 3). Sample labeling and charts are as described in legend of **Figure 5**, except: **(A**, **C**, **E)** tuber peel was collected at 60, 75, and 105 DATP, and **(B**, **D**, **F)** late in-season application of polyhalite (PolyH") was performed at 95 DATP and samples were collected at 105 DATP. Statistical analysis was performed as described for **Figure 5**.

Tuber peel samples of Vivaldi indicated increases in Ca and K concentrations between 60 and 75 DATP (**Figures 6A, E**), and a decline in Mg concentrations (**Figure 6C**), similar to the results for Rosanna. In contrast to Rosanna, a significant decline in Ca and K concentrations was then observed, which might have been due to the later sampling of Vivaldi, at 105 DATP compared to 95 DATP for Rosanna, due to local field practices. Similar to Rosanna, polyhalite mineral elements did not accumulate in the tuber peel following in-season application (**Figures 6B, D, F**). Overall, it can be concluded that polyhalite treatment prior to haulm desiccation did not alter the concentration of the tested mineral elements in the tuber peel.

**Figure 7:**
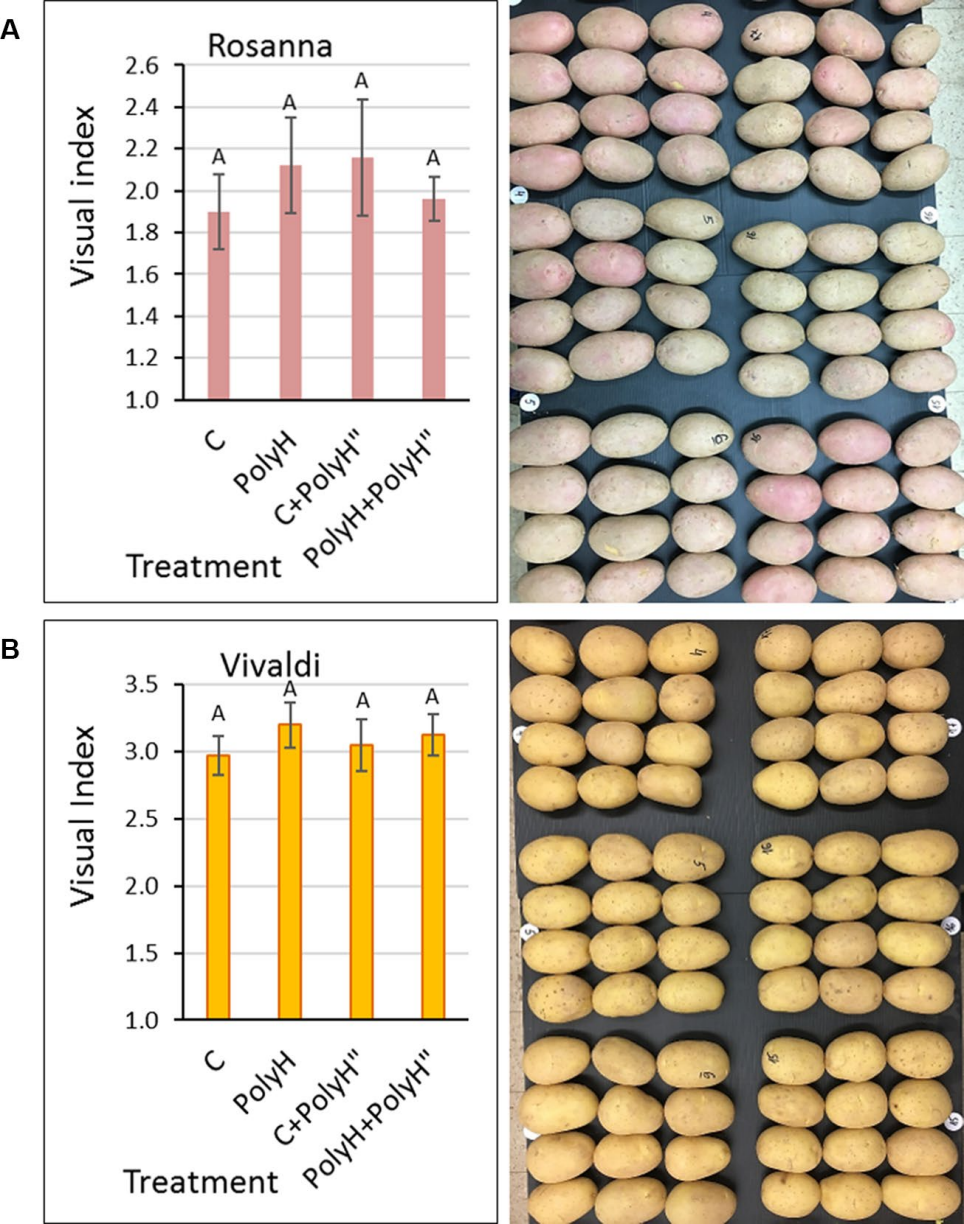
Evaluation of skin appearance of Rosanna **(A)** and Vivaldi **(B)** tubers from experiment 3. Sample labeling and treatments are as described in **Figures 5** and **6**. Visual evaluation of tuber appearance was performed after 2 months of storage (left panels). Values are averages of five replicates with SE. Visual index and assessment were as described in **Figure 2**. Data were not significant by Student’s *t*-test. Right panels demonstrate the problem of low skin quality and poor tuber appearance, irrespective of the polyhalite treatments. Rosanna tubers exhibited russeted skin with gray or faint pink color instead of the desired shiny appearance with dark-pink pigmentation. Tubers of Vivaldi exhibited russeted skin with brownish tint instead of the desired shiny yellow appearance.

Tuber appearance was examined after 2 months in storage (**Figure 7**). Skin quality was very poor for both Rosanna (**Figure 7A**) and Vivaldi (**Figure 7B**). The application of polyhalite either preplanting or in-season, or both, affected skin appearance positively, but not significantly.

### Skin Marker Gene Expression – Experiment 3

Although in-season application of polyhalite had only a minor effect on tuber appearance, data from experiment 1 in the previous year suggested an effect at the molecular level. Accordingly, the same genes were monitored in the tuber peels of Rosanna and Vivaldi, i.e., *KCS6*, *FHT*, and *CYP86A33*. Peel samples were collected from young tubers of Rosanna and Vivaldi at 75 DATP, when peel development activity is at its highest, and 10 days after the second application of polyhalite.

**Figure 8:**
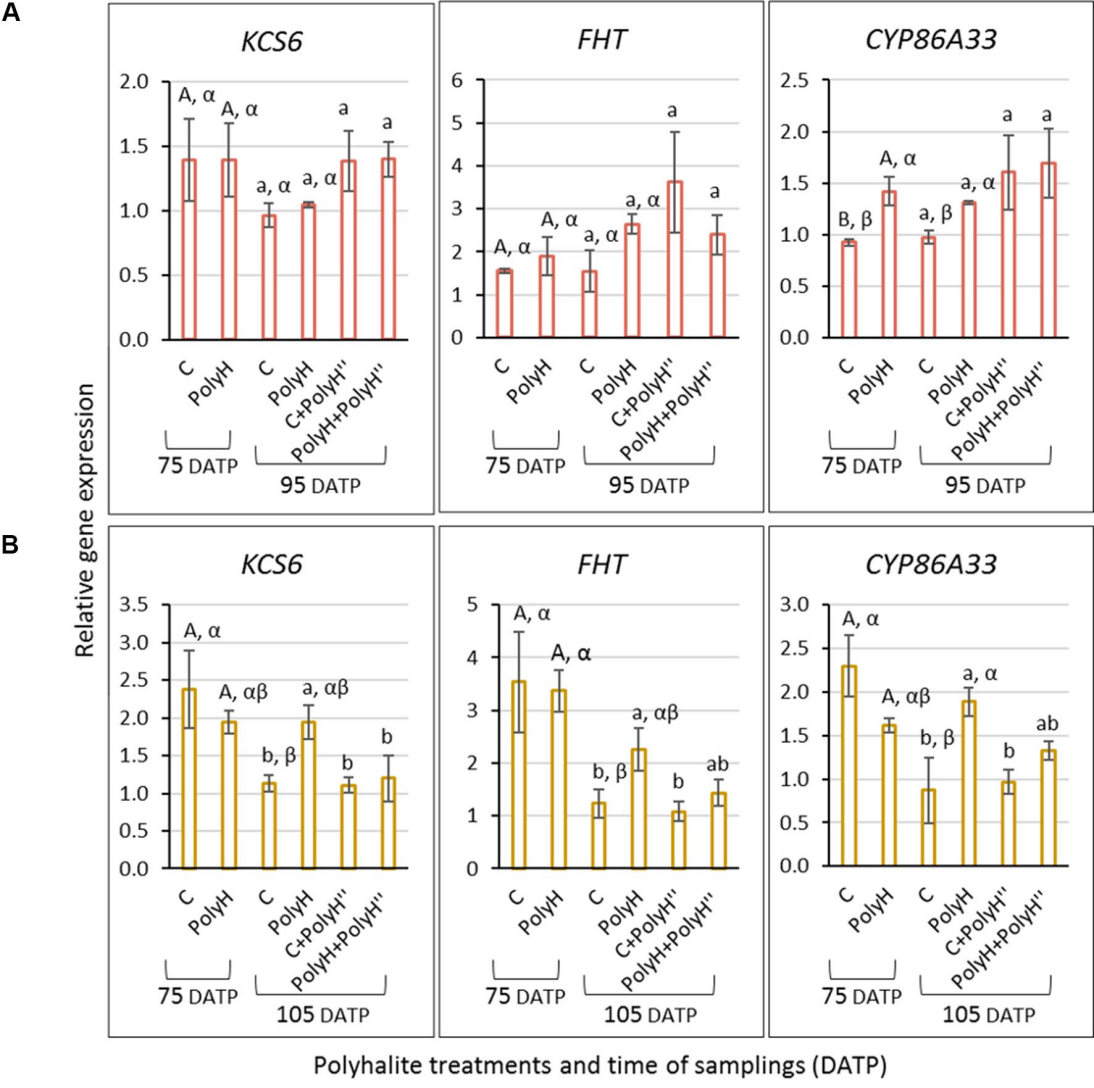
Expression of *KCS6*, *FHT*, and *CYP86A33* indicator genes for skin suberization in the peel of tubers from Rosanna **(A)** and Vivaldi **(B)** plants (experiment 3). Sample labeling and treatments are as described in **Figures 5** and **6**. Peel samples were collected from early-bulking tubers at 75 DATP, and 10 days after the late polyhalite application—95 DATP for Rosanna and 105 DATP for Vivaldi. Gene expression level and statistical analyses were performed as described in **Figure 3**. Uppercase letters compare samples from 75 DATP; lowercase letters compare samples from 95 DATP for Rosanna and 105 DATP for Vivaldi; Greek letters compare C and PolyH samples at the two time points.

For Rosanna (**Figure 8A**), in the peel of the young bulking tuber (75 DATP), the expression of *CYP86A33* was significantly higher in PolyH plants compared to controls, while for *FHT* expression trend was similar, albeit not significant. These genes exhibited the same expression trend when the peels were collected from maturing tubers at 95 DATP. No difference was found for *KCS6*. In-season application of the polyhalite, i.e., C+PolyH” and PolyH+PolyH” plants, all genes indicated increased expression compared to the control (sample C at 95 DATP); but it was not significant.

Interestingly, results for Vivaldi suggested different trend (**Figure 8B**). Gene expression in the peel of young plants (75 DATP) was significantly higher than in that from mature control plants (105 DATP), and higher than in the peel from the various applications of polyhalite. However, in maturing tubers at 105 DATP, the expression of all genes in the PolyH peel was higher than in the peel of the control.

### Anatomical Study of the Skin – Experiment 3

Histological analysis of tuber skin from both cultivars was conducted to gain additional data on the effect of polyhalite on skin development and quality. Samples included peel from young tubers at 60 DATP, and peels from maturing tubers at 95 DATP for Rosanna, or 105 DATP for Vivaldi.

**Figure 9:**
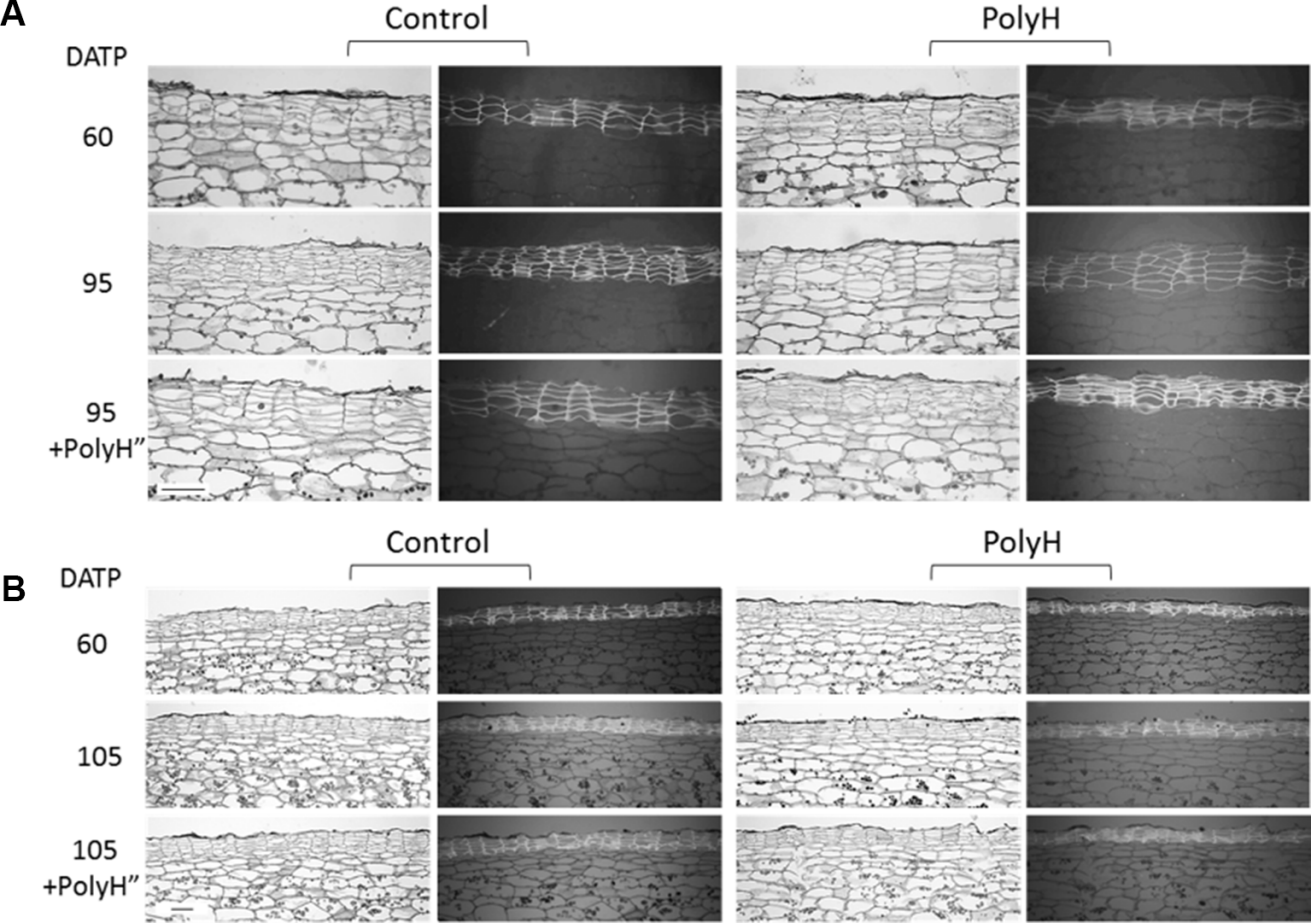
Anatomical study of tuber skin of potato cvs. Rosanna **(A)** and Vivaldi **(B)** (experiment 3). Sample labeling and treatments are as described in **Figures 5** and **6**. Sections were taken from three independent tubers for each treatment and developmental stage, and one representative section is shown. Each section is represented by two frames: the left shows tissue morphology under a light microscope, the right shows the autofluorescence of skin cells on a dark gray background as seen by UV microscope. Note that the morphology of the potato skin is characterized by cell column arrangements. Bar = 100 µm.

Both cultivars exhibited the characteristic skin anatomy, with columns of cells that autofluoresce under UV light (**Figure 9**). For both control and PolyH Rosanna plants, the peel of the maturing tubers (95 DATP) had a higher number of skin layers, which were also more condensed than in the skin of young tubers (60 DATP) (**Figure 9A**). The skin of mature PolyH tubers had bigger cells than the control; the skin cell layers of the latter seemed more condensed, as well. A second application of the polyhalite (PolyH”) resulted in increased skin cell size in control tubers, while the skin of PolyH+PolyH” tubers had condensed cell layers. Similar skin anatomy was observed for the Vivaldi tuber peel (**Figure 9B**).

## Discussion

### Mineral Fertilization and Tuber Peel Quality

In nonrusset potato cultivars, a rough appearance of the tuber peel, instead of being smooth and shiny, reduces potato marketability, not just for aesthetic reasons but also because this low-quality skin allows water loss from the tuber and is susceptible to skinning injuries.

Potato exhibits a strong interaction between genotype and the environment (Haynes et al., 2012), increasing tuber susceptibility to various physiological disorders, such as skin imperfections. One practice to combat skin problems is the application of divalent ions, such as those of Ca and Mg, which contribute to maintenance of the cell membrane and cell wall structure by forming stable but reversible linkages between the pectin polar head groups in the cell wall (Olsson 1988; Palta 1996). Positive effects of Ca (Ginzberg et al., 2012) and silica (Vulavala et al., 2016) on skin quality have also been demonstrated.

Most of the studies on nutrients fertilization of potato have focused on the quality of the tuber and its nutritional value. Ca concentration in the tuber flesh was shown to be enhanced by in-season application of Ca, even on soils that otherwise tested sufficient for exchangeable Ca (Gunter and Palta 2008). Transpiration is the main driving force for Ca transport. Being surrounded by moist soil, potato tubers have a very low transpiration rate, resulting in lower Ca per unit fresh weight than leaves (Palta 1996). This may be compensated for by the uptake of Ca from the soil solution *via* the roots on stolons and tubers (Kratzke and Palta 1985; Busse and Palta 2006). Accordingly, Ca fertilizer should be placed in the tuber area during the tuber-bulking period (Gunter and Palta 2008). Tuber acquisition of Ca has also been suggested to result from direct uptake from the soil across the periderm (Davies and Millard 1985; Subramanian et al., 2011). This was supported by the finding of high Ca in the skin compared to tuber flesh (Olsen et al., 1996; Subramanian et al., 2011)—the periderm contained 34% of the total tuber Ca concentration (Subramanian et al., 2011). However, direct transfer across the periderm can only happen at early stages of tuber development while the periderm is still a living tissue, before it becomes suberized (Habib and Donnelly 2002; Busse and Palta 2006). In mature tubers, there is no significant transport of Ca from the soil across the periderm (Busse and Palta 2006). Reports of higher Ca supply to the soil increasing the level of Ca in the tuber peel more than in its flesh (Olsen et al., 1996) imply that Ca fertilization results in its accumulation in the peel. In addition, the solubility of Ca fertilizers and Ca availability in the soil solution need to be considered (Olsen et al., 1996; Gunter and Palta 2008).

We previously showed that application of calcium chloride prior to tuber-seed sowing reduces the rate of tubers with russeted skin and the severity of the russeting (Ginzberg et al., 2012). Although the effect was statistically significant, potatoes with low skin quality and poor visual appearance are still a major problem in the industry. Here, polyhalite was tested as an alternative source of Ca, as we previously showed that it is a potent fertilizer than the equivalent soluble salts of Ca, Mg, and K (Yermiyahu et al., 2017). Moreover, transport and leaching of Ca, Mg, and K in the soil following polyhalite application was lower than following the application of the equivalent sulfate salts. The residual effect of polyhalite fertilizer on the subsequently grown crop is higher than the effect from the equivalent sulfate salts, especially with respect to Ca and Mg (Yermiyahu et al., 2017).

### Polyhalite Fertilization and Mineral Elements Concentration in the Peel During Tuber Development

The present data indicate that the pattern of mineral elements accumulation in tuber peel differs between Ca and K, and Mg. Furthermore, mineral elements acquisition by the peel of young bulking tubers (up to around 80–90 DATP) differs from that of maturing tubers (up to around 120 DATP), and that of tubers that are undergoing skin-set (after application of haulm desiccation). This was true for tuber peel from polyhalite-treated plants and the untreated controls. Combining the data from experiments 1 and 3, it is suggested that the peel of young developing tubers accumulates Ca and K at a high rate, which slows toward the end of the growth period (**Figures 5A, E** and **6A, E**), and strongly declines during skin-set (**Figures 1A, E**). The peel Ca concentration profile was in agreement with Olsen et al. (1996). Mg concentration in the peel was very low relative to Ca and K, and its concentration seemed to decline in young tubers (**Figures 5C** and **6C**) and slightly increase during skin-set (**Figure 1C**).

The effect of polyhalite application on mineral elements acquisition by the peel is not clear. When applied at the beginning of growth or in-season (3–4 weeks prior to haulm desiccation), there was no difference in mineral elements concentrations in the tuber peel between polyhalite-treated and control plants (**Figures 1A, C, E**, **5A, C, E**, and **6A, C, E**). However, late application of the polyhalite, after haulm desiccation, resulted in reduced concentrations of Ca and Mg and increased concentration of K (**Figures 1B, D, F**). We suggest that following haulm desiccation and induction of the skin-set process, the outer layer of the maturing skin consists of heavily suberized dead cells, i.e., corky material. The polyhalite mineral elements Ca, Mg, and K interact with this inert material as an ion exchanger, and K binding is favored over that of Ca and Mg (Shomer et al., 2003).

It can be argued that significant changes are not detected due to the low solubility of the polyhalite mineral, and thus a longer incubation time might reveal such changes. Alternatively, changes may be significant, but below detection levels. In addition, it may be assumed that polyhalite application affects the interactions between tuber peel and soil extract and thus affects tuber skin indirectly.

Although the motivation for this work was to increase available Ca, it is highly possible that Mg contributes to skin quality (Olsson 1988), and possibly K as well.

### Application of Polyhalite at the End of the Growth Period

External tuber skin cells are continuously sloughed off as the tuber expands. In parallel, new skin tissue is formed to cover the increasing surface area of the tuber. At the end of the growth period, tuber expansion stops, as well as skin formation. Practically speaking, the skin that is formed toward the end of tuber growth is the skin that covers the final potato product for marketing. Moreover, at the end of the growth period, the plant is aging and may not optimally support the final stages of skin maturation; this suggestion is backed by our observation of increasing incidence of skin imperfections when the growth period was extended beyond 120 DATP and plants seemed “exhausted”. Based on these considerations, we hypothesized that application of polyhalite to strengthen the skin of the final potato product should be performed at the end of growth. First, polyhalite was applied 2 days after haulm desiccation, assuming that the polyhalite mineral elements would support skin quality (Olsson 1988; Palta 1996) through the process of skin-set and skin maturation. Results indicated improved skin appearance (30%) of Rosanna potatoes compared to controls, regardless of whether polyhalite was additionally applied at the beginning of the growing period (**Figure 2A**, PolyH+PolyH”) or only once, after haulm desiccation (**Figure 2A, C**+PolyH”). This was more evident for potatoes after storage (**Figure 2B**). The visual index value of tubers from untreated plants was 30% lower than their value prior to storage, while that for PolyH+PolyH” tubers was reduced by only 10% following 1 month in storage, i.e., after storage, the visual index of PolyH+PolyH” tubers was 50% higher than that of the controls (**Figure 2B**).

Similar experiments with additional potato cultivars: Arizona, Sifra, Panamera, and Mozart, indicated improved tuber appearance of the first two, but reduced visual appearance for the latter two (**Figure 4**). Differences between cultivars may have resulted from variability in the genotype response to local growing conditions and to the polyhalite treatment. Nonetheless, it was concluded that polyhalite may improve tuber appearance when applied immediately after haulm desiccation.

Results were less conclusive when the polyhalite was applied 3–4 weeks prior to haulm desiccation (**Figure 7**). It was hypothesized that application of the polyhalite prior to periderm maturation might be more effective in improving periderm quality. Some improvement was obtained (**Figure 7**), but it was not significant, although the anatomical study indicated better organization of the skin cell columns, and possibly bigger phellem cells, following polyhalite application (**Figure 9**).

### Polyhalite Application Upregulates Skin-Suberizing Genes

The above results indicated some positive effect of polyhalite fertilization on tuber periderm characteristics, although they were not always significant. Hence, an expression study of genes known to be involved with suberization of potato skin (phellem cells) was conducted. The analyses were conducted for two experimental years and included *KCS6*, *CYP86A33*, and *FHT*. For the red-skinned cv. Rosanna, expression of *CYP86A33* and *FHT* was upregulated, albeit not significantly, in the periderm of young tubers following preplanting polyhalite fertilization (**Figure 8A**). Late application of polyhalite, shortly before or after haulm desiccation, resulted in these genes’ mostly significant upregulation (**Figure 8A** and **Figure 3**, respectively). No significant change was obtain in the expression of *KCS6*. Interestingly, the functions of KCS6 in fatty acid elongation (Serra et al., 2009a), and of CYP86A33 and FHT in suberin intramolecular cross-linking (Serra et al., 2009b; Serra et al., 2010), suggest that the polyhalite mineral affected mainly suberin cross-linking in the periderm of Rosanna.

Similar analysis in the periderm of the white-skinned cv. Vivaldi did not exhibit the same gene-expression profile as in Rosanna, and late application of polyhalite did not result in upregulation of the tested genes (**Figure 8B**). However, the expression of *KCS6*, *CYP86A33*, and *FHT* was significantly downregulated in 105-DATP periderm of control tubers when compared to young 75-DATP periderm. Preplanting polyhalite fertilization moderated this downregulation (**Figures 8B, C** and PolyH plants at 105 DATP). Overall, it was concluded that polyhalite application upregulates the expression of these skin-related genes.

### Concluding Comments

Our results indicate that polyhalite fertilization positively affects tuber skin appearance and skin-related gene expression. However, the effect was moderate and did not fully mitigate the skin imperfections. The causes for low skin quality are still unknown, and it may be that polyhalite mineral elements are not directly involved with the phenomenon. The high level of Ca in the local soil and irrigation water could also result in a moderate response, and it is assumed that the polyhalite effect will be more pronounced in soils that are poor in Ca and Mg. Finally, we note that other tuber and storage roots that are covered with corky periderm tissue like the potato tuber, including carrot and sweet potato, share similar skin problems (Villavicencio et al., 2007). Potato skin is the accepted model to study periderm development and skin imperfections/physiological blemishes, and the present study could be implemented in these important agricultural products as well.

## Data Availability Statement

The datasets generated for this study are available on request to the corresponding author.

## Author Contributions

AK-K and RB performed the anatomical and molecular analyses, EF collected the samples, IF carried out the mineral elements analyses, UZ conducted field experiments, UY and IG initiated the research project and planned the experiments, and IG carried out the literature review and wrote most of the manuscript.

## Funding

This research was supported by the Center for Fertilization and Plant Nutrition (CFPN), and is a contribution of ARO, the Volcani Center.

## Conflict of Interest

The authors declare that the research was conducted in the absence of any commercial or financial relationships that could be construed as a potential conflict of interest.
